# S02-3: Slovenian Approach to Enhance Physical Activity Among Vulnerable Populations

**DOI:** 10.1093/eurpub/ckae114.204

**Published:** 2024-09-26

**Authors:** Petra Ožbolt, Andrea Backović Juričan, Janja Križman Miklavčič

**Affiliations:** National Institute of Public Health, Slovenia; National Institute of Public Health, Slovenia; Ministry of Health, Slovenia

## Abstract

**Purpose:**

These policy documents foster physical activity and sports that empowers every individual, regardless of their age, health status, or background, to lead healthier, more active lives. Methodology was based on desk research, analysis and synthesis of Slovenian policy documents.

**Policy Description:**

In Slovenia, policy documents significantly influence the landscape of physical activity and sports, reflecting the nation's commitment to a healthier and more active society for all, including vulnerable populations.

The National Sports Program (2014-2023), outlines a strategic direction for sports development, with a focus on enhancing participation and excellence in sports. This program affects a wide range of populations, including children, adults, individuals with disabilities, and those with chronic diseases.

The National Nutrition and Physical Activity Programme (2015-2025), places strong emphasis on the importance of nutrition and physical activity in improving public health and overall well-being. The impact of this program extends to all citizens, with a particular emphasis on children, pregnant women, adults, and individuals with chronic diseases.

3. The Strategy for the Health of Children and Adolescents (2012; still ongoing), prioritizes the well-being of young generations by considering their environment, including opportunities for physical activity. This strategy primarily targets children and adolescents, addressing concerns related to obesity, health inequalities, and chronic non-communicable diseases.

The Action Programme for Persons with Disabilities (2022-2030), promotes accessibility to sports and physical activities for individuals with disabilities.

Strategic plan for the Development of the Cycling Network with Action Plan (Acceptance Predicted in 2024), aims to enhance active transportation options and promote physical activity, including people with disabilities.

The National Diabetes Prevention and Care Development Programme (2020-2030), have a significant impact on raising awareness about health and physical activity, primarily targets residents with diabetes and those at risk.

7. The National Cancer Control Programme (2022-2026), focusing on prevention and care.

**Conclusion:**

The impact of these policies extends to children, adults, and vulnerable populations, including those with disabilities and chronic non-communicable diseases. These policies create a framework that empowers all citizens, regardless of age or health status, to lead healthier, more active lives.

